# Musicogenic reflex seizure with positive antiglutamic decarboxylase antibody: A case report

**DOI:** 10.1002/epi4.12518

**Published:** 2021-07-21

**Authors:** Alawi A. Al‐Attas, Riyam F. Al Anazi, Saleh K. Swailem

**Affiliations:** ^1^ Department of Adult Neurology National Neuroscience Institute King Fahad Medical City Riyadh Kingdom of Saudi Arabia; ^2^ Department of Pediatric Neurology National Neuroscience Institute King Fahad Medical City Riyadh Kingdom of Saudi Arabia; ^3^ Department of Adult Neurology National Neuroscience Institute King Fahad Medical City Riyadh Kingdom of Saudi Arabia

**Keywords:** GAD‐Ab, musicogenic epilepsy, temporal lobe

## Abstract

The association of musicogenic epilepsy (ME) with antibodies against glutamic decarboxylase (GAD) supports autoimmune workups for these patients. No appropriate treatment has been established for ME; therefore, immunotherapy should be considered for patients who become drug‐resistant. The connection between neurological manifestations and antibodies against GAD, a rate‐limiting enzyme that helps create the inhibitory neurotransmitter gamma‐aminobutyric acid, has been well established. Furthermore, a strong correlation has been found between ME and the temporal lobe. However, its connection with anti‐GAD antibodies is still unclear. This paper reports on a 50‐year‐old right‐handed female who has had ME symptoms for 14 years and been found to be anti‐GAD antibody–positive. Therefore, we will elaborate on the relation between ME and anti‐GAD antibodies.

## 1 INTRODUCTION

Epilepsy is one of the leading neurological disorders, with a diagnosis requiring the presence of two unprovoked seizures >24 hours part. However, an International League Against Epilepsy (ILAE) task force has modified the definition of epilepsy to cope with exceptional circumstances that do not meet the two unprovoked seizure criteria.^1^ Reflex epilepsies are epileptic seizures that are consistently induced by an identifiable and objective‐specific trigger, which can be an afferent stimulus or the patient's activity. External stimuli range from simple light flash, fixation‐off, hot water, and visual, vestibular, auditory, and tactile triggers to complex stimuli such as reading or listening to music.^2^ Musicogenic epilepsy (ME) is a reflex seizure induced by sounds. It is rarely encountered, with a prevalence of 1 in 10 million people, but it has been reported since 1937.^3^ Autoantibodies against intracellular antigens such as glutamic acid decarboxylase (GAD) have been described mainly in patients with the clinical syndrome limbic encephalitis; however, these antibodies have also been described in patients with seizure alone such as ME, which supports an autoimmune workup in these patients.^4^ Glutamic acid decarboxylase is the principal enzyme that catalyzes the decarboxylation of the neurotransmitter glutamic acid to gamma‐aminobutyric acid (GABA). Neurological conditions, including stiff‐person syndrome, cerebellar ataxia, limbic encephalitis, myoclonus, and patients with epilepsy alone such as ME, have been linked to antibodies directed against GAD.^5^ Immunotherapy may be the primary treatment for patients who only show a partial response to antiepileptic drugs (AEDs).

## 2 CASE REPORT

Here, we report on a 50‐year‐old woman diagnosed with a seizure disorder for 14 years. She has a history of hypothyroidism and insulin‐dependent diabetes mellitus in treatment. The patient reported an aura with infrequent smells such as the odor of cooking food on an occasional basis. She has also been experiencing a recurrent loss of awareness associated with oral and left‐hand automatism, followed by generalized tonic‐clonic seizures. Each seizure episode tends to persist for a few seconds and does not have diurnal variations. The attacks tend to occur 4‐5 times per month, with the primary stimulus for her seizures being loud sounds such as particular music or songs; her seizures never occur spontaneously. Hence, she tries as much as possible to avoid such sounds. She has no history of CNS infection, no febrile seizures, no significant head trauma, and denies having any family member with a history of epilepsy. She has a normal perinatal history. The drugs that she has been using include control‐released carbamazepine (carbamazepine‐CR) 400 mg × q12h for the last 14 years, lamotrigine 100 mg × q12h for 14 years, and levetiracetam 1500 mg × q12h for 2 years. She also admits to having used valproate medication, but later stopped because she did not see its benefit.

The patient is a housewife and mother of five children. Her level of education is at the primary school level. Investigations showed her complete blood count, renal profile, hepatic profile, and thyroid function tests were normal; however, her HbA1c was elevated, and she was positive for anti‐GAD antibodies. Positron emission tomography and magnetic resonance imaging (MRI) of the brain were unremarkable.

**FIGURE 1:**
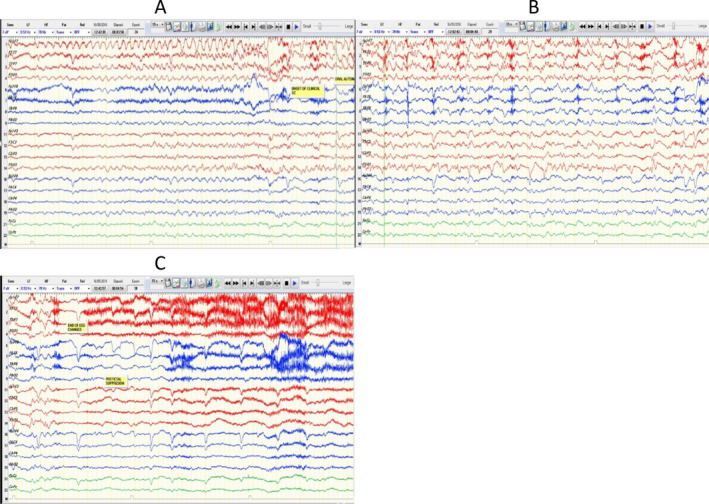
A‐C showing an ictal activity originating from the left hemisphere. An electrographic seizure with onset from the left temporal region. A,B, The seizure started with 5‐ to 6‐Hz lateralized rhythmic theta activity over left anterior temporal scalp electrodes, which has evolved in frequency and amplitude over the same area. C, The seizure ended by postictal focal slowing on the left temporal. The seizure started while the patient listening to the known trigger (Arabic song)

The neurological examination performed was normal. Later, she was admitted to the epilepsy monitoring unit for 14 days for a presurgical assessment. During admission, she experienced seven focal impaired‐awareness seizures, one of which ended with a focal‐to‐bilateral tonic‐clonic seizure. All attacks were triggered by listening to specific Arabic songs at a specific tone and were followed by a loss of awareness and oral and left‐hand automatism. She was assessed by and did not respond to the nurse who was present during the event's occurrence. An interictal electroencephalography (EEG) was characterized by normal posterior background activity of 8‐9 Hz during relaxed eyes‐closed wakefulness and demonstrated reactivity by decreasing amplitude and presence during eye‐opening. Sleep potentials were seen symmetrically; there were bitemporal independent sharp waves, more frequent in the left temporal area. Seizures were recorded from the left temporal lobe, although two were from the right temporal lobe (Figure 1). The epilepsy data include video‐EEG, and semiology indicates that this woman suffers from focal epilepsy triggered by listening to music (ME) arising from the left temporal lobe. The neuropsychology assessment showed a decline in her verbal memory, processing, and psychomotor speed. She performed above average on explicit episodic memory and nonverbal reasoning skills. Her other cognitive abilities were within the average range. Psychologically, she has been depressed, but no anxiety symptoms were reported. She has good social support and coping skills. On following up with the epilepsy clinic, the patient stated that she would have seizures only whenever she listens to Arabic music in a specific tone; hence, she preferred to avoid such music and song. The seizures are of the same type, and the frequency depends on the exposure to the triggers (song). She stated that she never has spontaneous seizures and does not agree to commence her on immunotherapy, and she did not report worsening in her cognition. As she refused immunotherapy in the form of IV immunoglobulin, she was advised to avoid the triggers and discharged on levetiracetam 1500 mg bid, carbamazepine‐CR 400 mg bid, and lamotrigine 100 mg bid.

## 3 DISCUSSION

Musicogenic epilepsy is a rare form of epilepsy induced by listening to or playing music. It was described by Critchley in early 1937 in his published paper “Musicogenic Epilepsy,” but in fact, the first probable communication is that of Merzheevsky in 1884, in which he reported on patients who had seizures provoked by melodies and musical tones.^6^ The provoking factors can be only from listening to music, as in our reported case; however, in some individuals, seizures can be provoked by both music and other nonauditory stimuli. Furthermore, the auditory triggers can be songs or specific portions of certain songs. Moreover, some ME cases can be induced by the triggering music's specific emotional content.^3^, ^7^ The latency can vary, with up to several minutes between the auditory trigger and the onset of the seizure. Indeed, the musical stimuli can range from simple tones to complex symphonic music.^8^ Using functional magnetic resonance imaging in 2005, Menon and Levitin observed a correlation between the activation of the nucleus accumbens and the ventral tegmental area, hypothalamus, and insula in response to music, which indicates connections between the emotional impacts of music and cognitive functions.^9^ Similar to the present case, Jesus et al presented a case study of a 61‐year‐old woman diagnosed with hypothyroidism, diabetes mellitus, and seizure disorder, with the seizures triggered by loud music and songs.^10^ Furthermore, Falip et al found that the hippocampus and insula are the main targets in patients with epilepsy and positive anti‐GAB antibodies. The article concludes that epilepsy combined with positive anti‐GAD antibodies unilaterally distresses the limbic system, and the effects frequently become bilateral.^11^ Lilleker et al conducted a literature review on the condition between 1884 and 2018 and found only two ME cases connected to anti‐GAD antibodies; no patient was reported to have stiff‐person syndrome. They concluded that the relation of anti‐GAD antibodies to epilepsy remains uncertain and did not support the routine use of immunotherapy in patients with epilepsy and GAD antibodies.^4^

Overall, ME is primarily evident in patients who have a temporal epileptogenic zone, and reportedly, it can originate from both dominant and nondominant hemispheres.^6^, ^12^, ^13^ In most ME cases, an emotional factor is described as the leading causal factor in the epileptogenic zone stimulation.^(2)^ First‐line treatment is avoiding the provocative triggers; if such measures and avoidance are not feasible, antiepileptic medication is another option. Alternatively, behavioral therapies, psychotherapy, and deconditioning techniques have been tried in those with high emotional states.^14^, ^15^ An autoimmune workup with an anti‐GAD antibody determination should be performed in patients with ME. In addition, a trial of immunotherapy may be considered in patients with drug‐resistant epilepsy and anti‐GAD antibodies. Surgery is another option in select cases of ME.^16^ Our patient chose to avoid the specific triggering music and remain on a AEDs. Hence, in the absence of the effects of immunotherapy on seizures, an immune pathogenesis in this case report cannot be confirmed.

## 4 CONCLUSION

Musicogenic epilepsy is a rare type of reflex epilepsy, where musical stimuli trigger seizures. Its association with anti‐GAD antibodies needs further elucidation. Immunotherapy may be considered in patients who fail preventive measures and become drug‐resistant. In select cases of ME, surgery may play role.

## CONFLICT OF INTEREST

None of the authors has any conflict of interest to disclose. We confirm that we have read the Journal's position on issues involved in ethical publication and affirm that this report is consistent with those guidelines.

## AUTHOR CONTRIBUTIONS

Alawi A. Al‐Attas conceived the case, collected the data, analyzed the data, created all the figures, and was primarily responsible for writing the manuscript. All authors participated in the clinical care and patient evaluations, and all authors reviewed the manuscript.

## ETHICAL APPROVAL

We confirm that we have read the Journal's position on issues involved in ethical publication and affirm that this report is consistent with these guidelines.
